# Endothelial Dysfunction in Adolescent Hypertension: Diagnostic Challenges and Early Cardiovascular Risk

**DOI:** 10.3390/jcdd12090326

**Published:** 2025-08-26

**Authors:** Vladimir Micieta, Michaela Cehakova, Ingrid Tonhajzerova

**Affiliations:** 1 Department of Physiology, Jessenius Faculty of Medicine in Martin, Comenius University in Bratislava, 036 01 Martin, Slovakia; 2 Institute of Medical Biology, Genetics, and Clinical Genetics, Faculty of Medicine, Comenius University, Sasinkova 4, 811 08 Bratislava, Slovakia

**Keywords:** adolescent hypertension, endothelial dysfunction, nitric oxide, oxidative stress, imaging, white-coat hypertension, masked hypertension, puberty, cardiovascular risk

## Abstract

Hypertension in adolescence causes early vascular injury manifesting as endothelial dysfunction (ED), which signifies elevated cardiovascular risk. This review synthesizes recent insights (2020–2025) into ED’s mechanisms and detection in hypertensive youth. We highlight how reduced nitric oxide bioavailability, oxidative stress, inflammation, and hormonal changes in puberty contribute to ED and consequent vascular remodeling. Non-invasive diagnostic tools (e.g., flow-mediated dilation, peripheral arterial tonometry) reveal that even asymptomatic hypertensive adolescents have measurable ED linked to arterial stiffness and cardiac changes. Encouragingly, ED in youth appears reversible: exercise and dietary interventions improve endothelial function, and pharmacotherapy (ACE inhibitors, ARBs) can restore endothelial health beyond blood pressure control. Early identification of ED in hypertensive adolescents is therefore critical—it not only refines risk stratification (e.g., unmasking high-risk “white-coat” hypertension) but also presents an opportunity to initiate lifestyle modifications and therapy to preserve vascular function.

## 1. Introduction

Adolescent hypertension is an increasingly prevalent condition that poses unique diagnostic and management challenges. While overt cardiovascular events are rare in youth, high blood pressure in adolescence is far from benign—it is associated with subtle but important early signs of target-organ damage. A critical mediator of this early damage is endothelial dysfunction (ED), defined as an impairment of the endothelium’s normal vasoregulatory and homeostatic functions, characterized by reduced vasodilation, a pro-inflammatory state, and a pro-thrombotic tendency. In adults, ED is a well-known precursor of atherosclerosis and correlates with future cardiovascular events. In the pediatric population, mounting evidence indicates that ED can be detected even in hypertensive children and adolescents, years before clinical cardiovascular disease becomes apparent [1,2,3,4,5,6,7,8,9,10,11].

This narrative review focuses on ED in adolescent primary (essential) hypertension. We examine the pathophysiology of ED in this context, its clinical relevance (including in white-coat and masked hypertension subtypes), and the tools available to diagnose ED in young patients. We also discuss how pubertal development and sex hormones may modulate endothelial function during adolescence. Finally, we explore current evidence on the reversibility of ED in youth—highlighting how early interventions (lifestyle or pharmacological) may improve endothelial function and potentially alter the trajectory of cardiovascular risk. Our goal is to identify diagnostic and therapeutic considerations that are particularly pertinent to hypertensive adolescents, a population in which preventing long-term vascular damage is paramount [12,13,14,15,16,17,18].

## 2. Materials and Methods

### Literature Search

**Figure 1:**
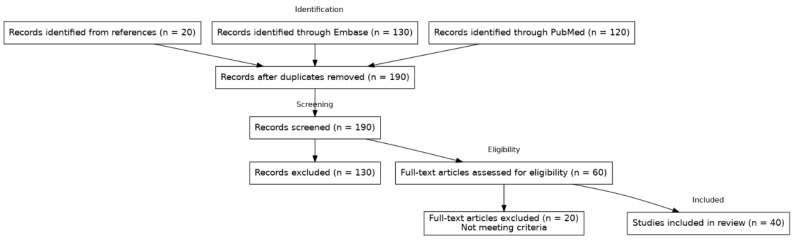
PRISMA Flow Diagram. PRISMA 2020 flow diagram of the literature search and selection process. Records were identified via PubMed (n = 120), Embase (n = 130), and by screening references (n = 20). Records after duplicates removed: n = 190. Records screened: n = 190. Records excluded: n = 130. Full-text articles assessed for eligibility: n = 60. Full-text articles excluded (not meeting criteria): n = 20. Studies included in review: n = 40.

The literature for this review was identified via PubMed and Embase searches (2020–2025) and by screening references of relevant articles; both clinical studies and reviews were included. Figure 1 illustrates the literature search and selection process according to PRISMA 2020 guidelines.

## 3. Clinical Relevance of Endothelial Dysfunction in Youth Hypertension

### 3.1. Endothelial Dysfunction as Early Indicator of Risk

In adults, the presence of endothelial dysfunction often heralds the development of atherosclerosis. Similarly, in hypertensive children and adolescents, ED serves as an early warning sign of vascular pathology. Studies have shown that adolescents with elevated blood pressure frequently exhibit impaired endothelium-dependent vasodilation (e.g., blunted flow-mediated dilation of the brachial artery) compared with normotensive peers [19]. These functional changes correlate with early structural changes: for instance, hypertensive youth tend to have increased arterial stiffness and greater carotid intima–media thickness (IMT) than normotensives of the same age [20,21,22]. Notably, such vascular changes are detectable even when hypertension is mild and before end-organ damage, like left ventricular hypertrophy (LVH), is clinically evident.

Because adolescents generally have healthy arteries otherwise, the detection of ED in this group strongly suggests that hypertension is already impacting the vasculature. In this sense, ED can be viewed as an integrative marker of cardiovascular risk—it reflects the cumulative burden of risk factors (blood pressure, obesity, etc.) on the vessel wall and portends future atherosclerotic disease. Recognizing ED in a hypertensive teenager is thus a signal to intensify efforts at risk-factor control.

### 3.2. “Target Organ” Implications—Heart and Vessels

Beyond being a risk marker, ED may actively contribute to the development of target-organ damage in young hypertensive patients. Endothelial dysfunction is accompanied by a pro-inflammatory and pro-thrombotic state, which over time can promote vascular remodeling. Impaired endothelial release of nitric oxide (NO) and excess oxidative stress (see Section 5.1) can lead to reduced arterial elasticity. Even early in the course of adolescent hypertension, pulse-wave velocity (a measure of arterial stiffness) is often elevated, indicating premature arterial aging. Stiffening of arteries increases the afterload on the heart, which may help explain why hypertensive adolescents can develop disproportionately high left-ventricular mass for their age.

Pediatric studies report that masked hypertension is associated with higher left-ventricular mass index and a greater prevalence of LVH on follow-up [23]. This illustrates how ongoing functional endothelial disturbance and persistent BP elevation can translate into structural cardiac changes.

### 3.3. White-Coat and Masked Hypertension: ED as a Differentiator

White-coat hypertension (WCH) and masked hypertension are common in pediatrics and carry differing risks. WCH refers to blood pressure that is elevated in clinic but normal outside. In referred pediatric cohorts, WCH prevalence has been reported in roughly 15–30% of children and adolescents who appear hypertensive on office readings. Historically, WCH was considered relatively benign, but more recent data complicate this view. Some adolescents with WCH show evidence of ED and subtle organ changes. For example, Jurko Jr. et al. observed that brachial FMD was reduced in WCH adolescents to a degree similar to that in sustained hypertensives (both significantly lower than normotensive controls) [24]. This suggests that a subset of WCH youth may have underlying vascular risk; ED testing might help identify which WCH patients have a profile closer to sustained hypertension. Conversely, if a WCH patient’s endothelial function is normal, that could reassure clinicians that the risk is lower—though such conclusions require further validation.

Masked hypertension, in contrast, is normal blood pressure in clinic but elevated at home or on ambulatory monitoring. It is less frequent (roughly 5–10% of adolescents) but is concerning because it can evade detection. Masked hypertension has been associated with a higher incidence of LVH and possibly more ED-related changes due to untreated elevated pressures. Adolescents with masked hypertension have been found to have higher ambulatory heart rates, more obesity, and, on echocardiography, higher left-ventricular mass than normotensives [25]. Identifying ED in WCH and masked hypertension could refine risk stratification: presence of ED in WCH might prompt closer monitoring or earlier intervention; in masked hypertension, ED would reinforce the need for treatment despite normal clinic readings.

## 4. Assessing Endothelial Function in Adolescent

### 4.1. Functional Tests: Brachial Flow-Mediated Dilation and Peripheral Arterial Tonometry

The two most commonly used clinical-research tools for assessing endothelial function in children are flow-mediated dilation (FMD) and peripheral arterial tonometry (PAT). Both are non-invasive and reflect endothelium-dependent vasodilatory capacity.

Flow-Mediated Dilation (FMD). This ultrasound-based test measures the percent dilation of the brachial artery during reactive hyperemia (usually provoked by inflation and release of a forearm cuff). A lower FMD percentage indicates worse endothelial function [26]. Absolute FMD cut-offs are not universally established in adults or children; methodological papers recommend standardized protocols and context-specific interpretation [26,27]. FMD is well-established in pediatric research; for example, one study noted that hypertensive adolescents had significantly lower FMD compared with controls (hypertensive children had a median FMD of ~5.8% vs. ~8.3% in normotensive controls). Additionally, FMD was inversely correlated with carotid IMT and ambulatory BP load in these youths, suggesting functional impairment goes hand-in-hand with structural and hemodynamic burden [28]. Thus, FMD provides a direct gauge of conduit–artery health and has been linked to early target-organ changes in hypertension.

Peripheral Arterial Tonometry (PAT). This method (e.g., EndoPAT device) uses finger plethysmographic probes to measure pulse–volume changes during reactive hyperemia. The output is the reactive hyperemia index (RHI)—a ratio indicating the degree of microvascular dilator response in the finger. PAT is attractive for adolescent studies because it is operator-independent and easy to perform. Studies show that conventional RHI sometimes fails to distinguish mild hypertensive from normotensive teens [29]. To improve sensitivity, novel PAT-derived indices have been proposed. Jurko et al. (2022) introduced the hyperemic area-under-the-curve (AUC) of the PAT signal as an enhanced measure and found that it differentiated sustained hypertensives from others even when RHI did not [30,31]. This suggests that analyzing the time-course of the hyperemic response [27] can reveal microvascular ED in youth with sustained hypertension.

Summary. FMD and PAT (with advanced analysis) are valuable, non-invasive tools. Impaired brachial FMD or a blunted PAT hyperemic response in an adolescent supports the presence of ED and can be tracked over time or after interventions.

### 4.2. Non-Invasive Vascular Imaging in Adolescents

Beyond functional tests, several imaging modalities can assess early vascular changes associated with ED in adolescents. Carotid ultrasonography can measure IMT; elevated IMT in youth has been linked to hypertension and dyslipidemia. Pulse–wave velocity (PWV), measured by tonometry or oscillometric devices, provides an index of arterial stiffness and is often elevated in hypertensive adolescents, signifying premature arterial aging. Echocardiography can detect cardiac effects such as increased LV mass or diastolic dysfunction associated with longstanding high BP. Even the retinal vasculature may reflect hypertensive changes in severe cases. These imaging assessments complement functional tests: while FMD/PAT evaluate endothelial function, imaging demonstrates structural consequences (arterial stiffening, cardiac remodeling). Combining functional and structural assessments provides a more comprehensive picture of vascular health in hypertensive adolescents. (Table 1 in the manuscript summarizes representative studies on ED in hypertensive youth).

## 5. Pathophysiology and Biomarkers of ED in Adolescent Hypertension

### 5.1. Nitric Oxide and Oxidative Stress

Endothelial-derived nitric oxide (NO) is a key vasoprotective molecule. In ED, decreased NO bioavailability is a central feature. During normal adolescence, rising levels of sex steroids and growth hormones can modulate vascular tone and endothelial function [19], but in hypertensive teens these effects may be blunted by oxidative stress and vascular load, and excess weight is associated with increased arterial stiffness [20]. Mechanistically, hypertension-related neurohumoral activation and oxidative stress reduce NO signaling and promote vasoconstriction; comprehensive reviews detail these pathways [35]. Biomarkers of oxidative stress can be elevated, whereas antioxidant capacity may be reduced [35]. Asymmetric dimethylarginine (ADMA)—an endogenous inhibitor of eNOS—is often higher in pediatric hypertension and obesity [36,37]. Importantly, NO bioavailability appears modifiable: in a pediatric trial, ramipril markedly reduced ADMA and inflammatory markers, suggesting a direct endothelial benefit beyond BP lowering [33,38]. Overall, reduced NO bioavailability is a central abnormality in hypertensive ED.

### 5.2. Inflammation and Endothelial Activation

Hypertension is accompanied by low-grade inflammation. Endothelial cells in a pro-inflammatory environment express higher levels of adhesion molecules and release cytokines, further propagating vascular injury; inflammation–ED interactions in pediatric hypertension are summarized in recent reviews [35]. Circulating inflammatory markers (e.g., high-sensitivity CRP, IL-6, TNF-α) may be elevated. Microparticles (extracellular vesicles) shed from activated endothelium can also be increased. Novel biomarkers include endocan, which correlates with ED severity in obese pediatric patients [34].

### 5.3. Pubertal Modulation of Endothelial Function

Adolescence is a time of significant hormonal and metabolic changes that can transiently impact endothelial function. In healthy youths, endothelium-dependent vasodilation tends to improve through puberty, likely as a result of hormonal changes [19]. In the context of hypertension, these beneficial effects may be diminished. Sex and pubertal stage can modulate cardiovascular risk markers in youth [19]; clinicians should consider the developmental stage when evaluating ED in adolescent hypertensives.

## 6. Reversibility of Endothelial Dysfunction and Therapeutic Interventions

**Lifestyle.** Multiple studies show that regular aerobic exercise and weight reduction benefit endothelial function in children and adolescents; improvements are seen in FMD and arterial stiffness, with larger and longer programs producing greater gains [32,37,39].

**Pharmacotherapy**. When lifestyle measures are insufficient, antihypertensives not only reduce BP but may also improve endothelial function. ACE inhibitors are of particular interest: in a randomized pediatric trial, ramipril significantly reduced ADMA and inflammatory cytokines independently of BP changes [33]. Earlier intervention may attenuate vascular remodeling and stiffness progression in youth [40].

**Combined strategies**. The greatest improvements likely arise when lifestyle optimization is paired with rational pharmacotherapy. Given the apparent reversibility of ED in youth, timely detection and intervention may normalize endothelial function and improve long-term cardiovascular outlook.

## 7. Conclusions

Adolescent hypertension is frequently accompanied by endothelial dysfunction—an early sign of vascular injury that precedes clinical disease. ED refines risk stratification (including in white-coat and masked hypertension) and offers a modifiable target. Non-invasive tests such as FMD and PAT, along with complementary imaging and biomarkers, enable early detection. Because ED in youth is often reversible, prioritizing lifestyle measures and, when indicated, pharmacotherapy can improve endothelial function and help preserve long-term cardiovascular health.

## Figures and Tables

**Table 1:** Summary of Selected Studies on Endothelial Function in Hypertensive Youth.

Study (Year)	Population/Design	Key Findings (Endothelial Function and Outcomes)
**Lurbe et al., 2013** *[13]*	Referred children and adolescents; ABPM-based classification; cross-sectional prevalence (white-coat hypertension).	Reported prevalence and determinants of white-coat hypertension in youth; highlights need for ABPM to avoid misclassification; WCH associated with higher office BP and adiposity.
**Jurko Jr. et al., 2018** *[24]*	~90 adolescents (cross-sectional)—groups: sustained HTN, white-coat HTN, normotensive controls. Endothelial function by brachial FMD.	FMD was significantly lower in both sustained hypertensive and white-coat hypertensive adolescents compared with controls (*p* < 0.05 for both). No difference between white-coat vs. sustained HTN FMD, suggesting even WCH had comparable endothelial dysfunction to sustained HTN. Supports that WCH is not entirely benign in youth.
**Jurko et al., 2022** *[30]*	100 adolescents (mean ~15 y)—normotensive (~34), essential HTN (~33), white-coat HTN (~33). Endothelial function measured by PAT (RHI and novel hyperemia indices).	Essential HTN had lower PAT hyperemic AUC index than normotensive and WCH groups (*p* = 0.02–0.03). Conventional RHI did not differ, but AUC was sensitive to ED in sustained HTN. AUC inversely correlated with mean BP (r = –0.23) and positively with pubertal hormone (DHEA). White-coat HTN had AUC similar to normals, indicating preserved microvascular function in WCH (contrast to macrovascular FMD findings). First study to show microvascular ED via PAT in adolescent HTN.
**Doğan et al., 2024** *[28]*	40 adolescents (~16 y; 20 newly diagnosed essential HTN without treatment, 20 healthy controls). Cross-sectional measurement of carotid IMT, brachial FMD, and capillary density (nailfold capillaroscopy).	Hypertensive youth had higher carotid IMT (mean diff *p* = 0.04), lower FMD (HTN ~5.8% vs. control ~8.3%, *p* = 0.02), and reduced functional capillary density (*p* < 0.001) compared with controls. No difference between dipper vs. non-dipper HTN subgroups. Conclusion: Essential HTN is associated with increased arterial stiffness/thickness and significant endothelial dysfunction in adolescents, emphasizing the need for early management.
**Watts et al., 2004** *[32]*	Obese adolescents (n = 19; 14.3 ± 1.5 y), randomized crossover; 8-week supervised circuit training vs. habitual activity; lean controls (n = 20) for baseline comparison; conduit-artery endothelial function by brachial FMD; body composition by DXA	Baseline FMD impaired vs. lean controls (5.3 ± 0.9% vs. 8.9 ± 1.5%); after 8 weeks of training FMD normalized (8.8 ± 0.8%); abdominal and trunk fat decreased; fitness and muscular strength improved (all *p* < 0.05)
**Ateya et al., 2022** *[33]*	135 children (7–15 y) on maintenance hemodialysis with hypertension. Randomized placebo-controlled trial: Ramipril 2.5 mg QD (n = 68) vs. placebo (n = 67) for 16 weeks. Primary endpoints: plasma ADMA (endothelial dysfunction marker) and hs-CRP; secondary: IL-6, TNF-α, BP.	Ramipril group showed significant improvements in endothelial biomarkers: ADMA decreased by 80%, hs-CRP by 46%, IL-6 by 27%, TNF-α by 52% (all *p* < 0.001 vs. baseline; significantly greater reductions than placebo). BP fell in both groups, but more in Ramipril (−12/−9 mmHg vs. placebo). Notably, reductions in ADMA and inflammatory markers did not correlate with BP changes, suggesting a direct endothelial benefit of ACE inhibition. **Implication:** ACE inhibitors restore endothelial function (improve NO availability and lower inflammation) in children, beyond their BP-lowering effect.
**Dias et al., 2015** *[34]*	Meta-analysis of randomized exercise interventions in overweight/obese children and adolescents (aggregate N = 219); primary outcome: brachial FMD; secondary: VO_2_peak.	Exercise training vs control improved FMD (mean difference + 1.54 % absolute, *p* < 0.05) and increased VO_2_peak (+3.64 mL·kg^−1^·min^−1^, *p* < 0.05).

## Data Availability

No new data were created or analyzed in this study.
